# A Predictive Model for the 10-year Overall Survival Status of Patients With Distant Metastases From Differentiated Thyroid Cancer Using XGBoost Algorithm-A Population-Based Analysis

**DOI:** 10.3389/fgene.2022.896805

**Published:** 2022-07-08

**Authors:** Shuai Jin, Xing Yang, Quliang Zhong, Xiangmei Liu, Tao Zheng, Lingyan Zhu, Jingyuan Yang

**Affiliations:** ^1^ School of Big Health, Guizhou Medical University, Guiyang, China; ^2^ School of Medicine and Health Administration, Guizhou Medical University, Guiyang, China; ^3^ Department of Urology, The Affiliated Hospital of Guizhou Medical University, Guiyang, China; ^4^ School of Clinical Medicine, Guizhou Medical University, Guiyang, China; ^5^ Health Management Center, The Affiliated Hospital of Guizhou Medical University, Guiyang, China; ^6^ School of Public Health, Guizhou Medical University, Guiyang, China

**Keywords:** differentiated thyroid cancer, Xgboost algorithm, machine learning, distant metastases, predictive model, SEER database

## Abstract

**Purpose:** To explore clinical and non-clinical characteristics affecting the prognosis of patients with differentiated thyroid cancer with distant metastasis (DTCDM) and establish an accurate overall survival (OS) prognostic model.

**Patients and methods:** Study subjects and related information were obtained from the National Cancer Institute’s surveillance, epidemiology, and results database (SEER). Kaplan‐Meier analysis, log-rank test, and univariate and multivariate Cox analysis were used to screen for factors influencing the OS of patients with DTCDM. Nine variables were introduced to build a machine learning (ML) model, receiver operating characteristic (ROC) was used to evaluate the recognition ability of the model, calibration plots were used to obtain prediction accuracy, and decision curve analysis (DCA) was used to estimate clinical benefit.

**Results:** After applying the inclusion and exclusion criteria, a total of 3,060 patients with DTCDM were included in the survival analysis from 2004 to 2017. A machine learning prediction model was developed with nine variables: age at diagnosis, gender, race, tumor size, histology, regional lymph node metastasis, primary site surgery, radiotherapy, and chemotherapy. After excluding patients who survived <120 months, variables were sub-coded and machine learning was used to model OS prognosis in patients with DTCDM. Patients 6–50 years of age had the highest scores in the model. Other variables with high scores included small tumor size, male sex, and age 51–76. The AUC and calibration curves confirm that the XGBoost model has good performance. DCA shows that our model can be used to support clinical decision-making in a 10-years overall survival model.

**Conclusion:** An artificial intelligence model was constructed using the XGBoost algorithms to predict the 10-years overall survival rate of patients with DTCDM. After model validation and evaluation, the model had good discriminative ability and high clinical value. This model could serve as a clinical tool to help inform treatment decisions for patients with DTCDM.

## Introduction

Differentiated thyroid cancer (DTC) is the most common endocrine malignancy, with the global incidence increasing dramatically in recent decades (Cabanillas et al., 2016; Siegel et al., 2020). Most DTC patients have a good long-term prognosis because of biological characteristics and effective responses to treatment modalities (Lim et al., 2017; Liu et al., 2018). For TC patients with distant metastasis (DM), however, the overall prognosis is significantly worse (Durante et al., 2006; Sugitani et al., 2008; Farooki et al., 2012; Nies et al., 2021).

The main histological subtypes of DTC include papillary thyroid carcinoma (PTC), and follicular thyroid carcinoma (FTC), <10% of people with DTC will develop DM (Durante et al., 2006). Most DTCDM is asymptomatic and only detected during systematic surveillance or systemic metastatic examination of malignant lymph nodes. The common site of distant metastases from thyroid cancer is the lung, followed by the bone, brain, and liver (Chesover et al., 2021; Liu et al., 2021). Because the incidence of DM is low and there is often an absence of symptoms, it is frequently overlooked at the time of initial TC diagnosis. Ten-year survival rates are often used to assess DTC treatment efficacy and characterize risk factors. For patients with metastatic thyroid cancer, treatment is often individualized and usually consists of thyroid surgery with adjuvant radiotherapy or chemotherapy (Sampson et al., 2007). While prognostic models have been developed to study factors influencing different primary cancer subtypes (Zhao et al., 2019; Zhou et al., 2020; Jin et al., 2021; Kong et al., 2021), the 10-years overall survival of patients with DTCDM is unclear, especially because OS has not changed significantly in recent decades (Goffredo et al., 2013). In addition, there are few prognostic models for DTCDM that can help to inform patient follow-up and treatment decisions (Chen et al., 2021).

Machine learning (ML) is an emerging multi-disciplinary approach used to correlate multiple discrete variables and accurately predict outcomes. Following the development of evidence-based medicine and the need for more advanced tools to collect medical data with complex structures and large sample sizes, ML emerged as an alternative approach to disease diagnosis and prognosis, with high predictive performance and a wide range of applications (Goecks et al., 2020; May, 2021). ML algorithms are now being successfully applied to predict cancer survival (Angraal et al., 2020). The XGBoost algorithm (XGB), in particular, is shown to have excellent prediction performance in previous studies (Senders et al., 2020; Jiang et al., 2021).

The Surveillance, Epidemiology, and End Results (SEER) program is a population-based cancer registry system sponsored by the United States’ National Cancer Institute (NCI) that currently covers about 28% of the population in 18 registered states (Noone et al., 2016). Using the considerations listed above, a prognostic modeling analysis of patients with DTCDM was conducted using SEER data. This study assessed the ability of clinical and non-clinical factors to predict DTCDM using the XGB model. The XGB model was also built to predict the 10-years OS rate of TC patients with DM. The performance of the XGBoost model using logistic regression (LR), random forests (RF), and support vector machine (SVM) models were compared.

## Materials and Methods

### Study Population

This study used the SEER database (https://seer.cancer.gov/) developed by the National Cancer Institute, a free cancer registry in the United States. A data use agreement was signed with the SEER database official and all authors followed the specified conditions. Because SEER data is freely available to researchers and patient personal information is officially withheld, no moral or ethical support from the host institution was required for this study.

**FIGURE 1:**
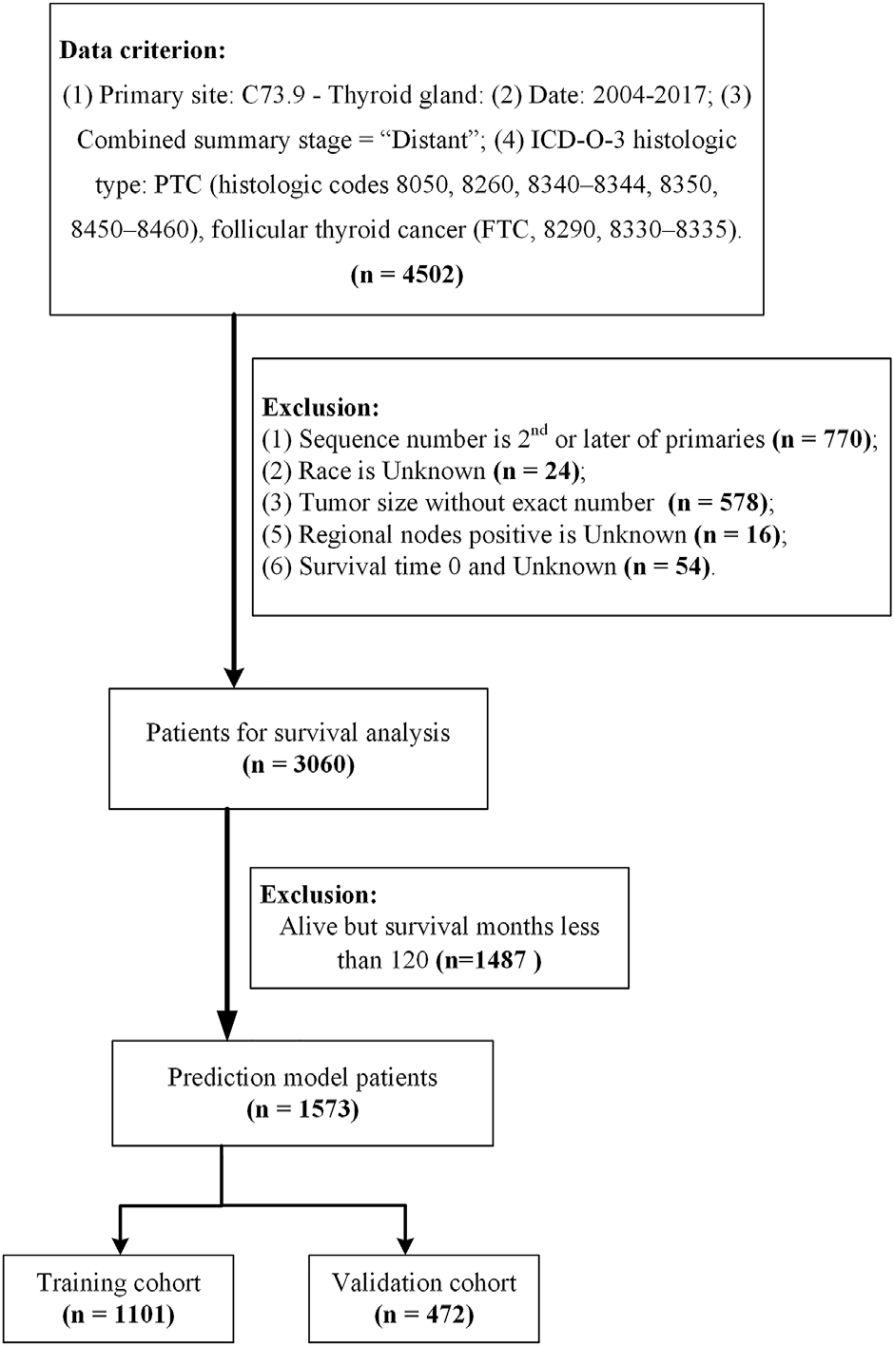
Sample screening process.

Data was downloaded from the SEER 18 regs plus database using SEER*Stat (Version 8.3.9.2, https://seer.cancer.gov/data-software/). Data were selected from patients with histologically confirmed distant metastatic thyroid cancer using the following criteria: 1) primary site code is C73.9 - thyroid gland, 2) dates ranging from 2004 to 2017, 3) PTC (histologic codes 8050, 8260, 8340–8344, 8350, 8450–8460), FTC (8290, 8330–8335), and 4) diagnosis confirmation combined with a summary stage for the distant future. Exclusion criteria included 1) sequence numbers for second or later occurrences, 2) unknown race, 3) unclear tumor size, 4) unknown surgery, 5) unknown regional nodes positive (RN_positive), and 6) survival time of 0 or unknown. A total of 3,060 patients were included in the survival analysis to clarify possible factors influencing the prognostic model. Of these, 1,487 patients who had survived <120 months by the follow-up cut-off date were excluded and 1,573 patients were included in the prediction model and grouped in the training set (*n* = 1,101; 70%) or the validation set (*n* = 472; 30%) at a 7:3 ratio **(** Figure 1).

### Variable Selection and Endpoints

To take full advantage of ML, several established demographic and clinical characteristics were selected as independent variables for analysis. Pathology variables commonly used in thyroid cancer research, including tumor size, histological type, regional lymph node status, surgical modality, radiotherapy, and chemotherapy, and demographic indicators, including age, gender, and ethnicity were included. Age and tumor size were optimally stratified for processing using X-tile software (https://medicine.yale.edu/lab/rimm/research/software/) and included in the survival analysis. These analyses were performed before excluding patients who survived for <120 months. The 10-years OS rate for patients with DTCDM was defined as the model endpoint.

### Statistical Analysis

All statistical analyses and model building in this study were performed using R (version 4.1.2, https://www.r-project.org/). The Kaplan–Meier method, with both univariate and multifactorial Cox, was used to screen for OS prognostic factors in thyroid cancer patients, and all variables were used to construct prognostic models. The Chi-square test was then used to analyze differences between the training and validation cohorts. The training set was used to build the XGBoost model and the model was evaluated with the test set. The model capability evaluation includes the following three items: 1) receiver operating characteristic (ROC) curves were used to analyze model discrimination and the area under the ROC curve (AUC) was used to assess predictive model accuracy (Hanley and McNeil, 1982; Wolbers et al., 2009); 2) calibration plots were used to assess the performance of the model, which calibrates how well model predictions agree with the actual observations (Leonard et al., 2020); and 3) decision curve analysis (DCA) was used to assess the clinical usefulness and net benefit of predictive model performance by calculating the difference between the true and false positive rates, weighted by the probability of the chosen risk threshold to assess the net benefit of the model (Vickers and Elkin, 2006). Logistic regression, SVM, and random forest models were built for comparison to the XGBoost model.

## Results

### Baseline Characteristics and Survival Analysis

**FIGURE 2:**
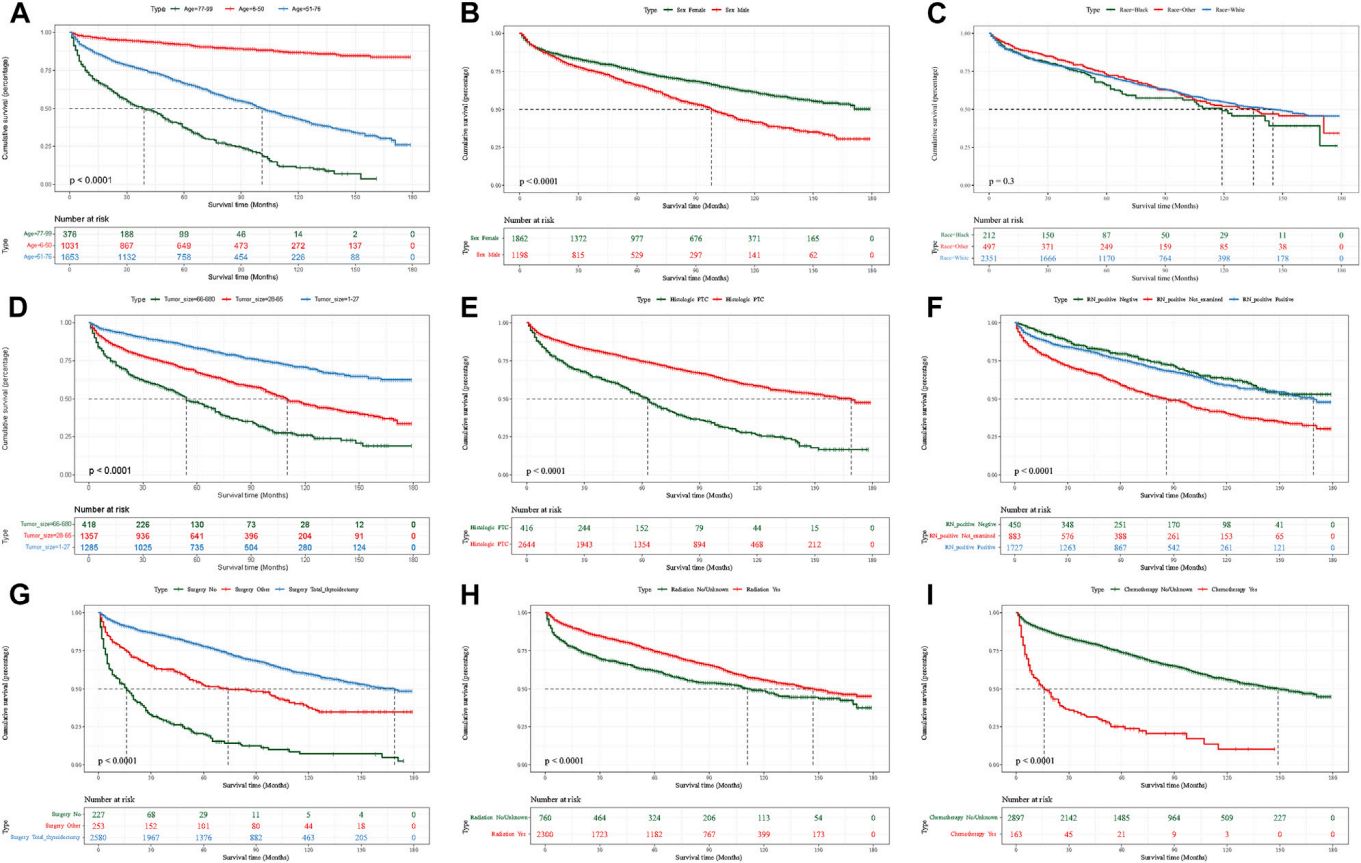
Kaplan–Meier survival curves to evaluate the impact of each classified characteristic.

A total of 3,060 patients with DTCDM were included in the survival analysis for variable screening. The best cut-off values for age were 50 and 76 years old, and the tumor size was 27 and 65 mm (Supplementary Figure S1). Most of the study population (54.02%) was 51–76 years of age with a higher proportion of females than males. Most patients’ tumor size was <6.5 cm and the most common histological type was PTC (86.41%), and FTC type has about 14%. Above half of patients had regional node metastases (56.44%) and total thyroidectomy, radiotherapy, and chemotherapy were used for 56.44%, 76.16%, and 5.33% of patients, respectively. After validation using the Kaplan–Meier method and log-rank test, only survival rates of DTCDM patients did not differ between races (Figure 2C), and all other variables differed at all scales (Figures 2A,B,D–I). We performed univariate and multivariate Cox models for age, gender, race, tumor size, histological type, RN_positive, surgery, radiotherapy, and chemotherapy, respectively. The multivariate model showed that all variables were independent prognostic factors for DTCDM, except for the race **(** Table 1).

**TABLE 1:** The baseline characteristics, univariate and multivariate cox analysis.

Characteristics	Total	Univariate		Multivariate	
	(*n*, %)	HR (95% CI)	*p*	HR (95% CI)	*p*
Age at diagnosis, years					
77–99	376 (12.29)	References		References	
6–50	1031 (33.69)	0.07 (0.06–0.09)	<0.001	0.10 (0.08–0.13)	<0.001
51–76	1653 (54.02)	0.41 (0.36–0.47)	<0.001	0.49 (0.43–0.57)	<0.001
Sex					
Female	1862 (60.85)	References		References	
Male	1198 (39.15)	1.59 (1.41–1.79)	<0.001	1.27 (1.12–1.43)	<0.001
Race					
Black	212 (6.93)	References		References	
Other	497 (16.24)	0.82 (0.64–1.07)	0.141	0.98 (0.75–1.27)	0.858
White	2351 (76.83)	0.85 (0.68–1.06)	0.143	1.04 (0.83–1.31)	0.714
Tumor size, mm					
66–680	418 (13.66)	References		References	
28–65	1357 (44.35)	0.53 (0.46–0.61)	<0.001	0.7 (0.70–0.82)	<0.001
1–27	1285 (41.99)	0.25 (0.21–0.29)	<0.001	0.41 (0.34–0.49)	<0.001
Histologic					
FTC	416 (13.59)	References		References	
PTC	2644 (86.41)	0.42 (0.36–0.48)	<0.001	0.78 (0.66–0.91)	<0.001
RN positive					
Negative	450 (14.71)	References		References	
Not examined	883 (28.86)	2.08 (1.71–2.52)	<0.001	1.34 (1.09–1.64)	0.005
Positive	1727 (56.44)	1.15 (0.95–1.39)	0.145	1.47 (1.21–1.79)	<0.001
Surgery					
No	227 (7.42)	References		References	
Other	253 (8.27)	0.33 (0.26–0.41)	<0.001	0.53 (0.42–0.67)	<0.001
Total thyroidectomy	2580 (84.31)	0.16 (0.13–0.18)	<0.001	0.35 (0.28–0.42)	<0.001
Radiation					
No	760 (24.84)	References		References	
Yes	2300 (76.16)	0.66 (0.58–0.75)	<0.001	0.86 (0.75–0.98)	0.029
Chemotherapy					
No	2897 (94.67)	References		References	
Yes	163 (5.33)	4.73 (3.89–5.75)	<0.001	2.55 (2.07–3.13)	<0.001

RN, positive, regional nodes metastases.

### Prognostic Model Construction

Using survival analysis, the following nine variables were included in the prognostic model: age, sex, race, tumor size, histological type, RN_positive, surgery, radiation, and chemotherapy. Most patients (65.9%) with DTCDM ≥10 years were 51–76 years of age, and a higher proportion were women (59.0%) and had PTC histologic staging (81.8%), total thyroidectomy (76.7%), radiotherapy (72.3%), and chemotherapy (7.6%). Analysis of the differences between the two groups for each variable showed no statistical difference, indicating good comparability between the training and validation sets **(** Table 2).

**TABLE 2:** The baseline characteristics of the training and validation sets used in the prognostic model.

Characteristics	Total cohort	Training cohort	Validation cohort	χ^2^	*p*
	*n* (%)	*n* (%)	*n* (%)		
	1573 (100.0)	1101 (70.0)	472 (30.0)		
Age at diagnosis, years				2.011	0.366
77–99	281 (17.9)	202 (18.3)	79 (16.7)		
6–50	375 (23.8)	252 (22.9)	123 (26.1)		
51–76	917 (58.3)	647 (58.8)	270 (57.2)		
Sex				0.003	0.959
Female	928 (59.0)	650 (59.0)	278 (58.9)		
Male	645 (41.0)	451 (41.0)	194 (41.1)		
Race				1.895	0.388
Black	108 (6.9)	81 (7.4)	27 (5.7)		
Other	256 (16.3)	183 (16.6)	73 (15.5)		
White	1209 (76.9)	837 (76.0)	372 (78.8)		
Tumor size, mm				0.752	0.687
66–680	273 (17.4)	197 (17.9)	76 (16.1)		
28–65	745 (47.4)	519 (47.1)	226 (47.9)		
1–27	555 (35.3)	385 (35.0)	170 (36.0)		
Histologic				1.961	0.161
FTC	286 (18.2)	210 (19.1)	76 (16.1)		
PTC	1287 (81.8)	891 (80.9)	396 (83.9)		
RN positive				0.18	0.914
Negative	220 (14.0)	154 (14.0)	66 (14.0)		
Not examined	585 (37.2)	413 (37.5)	172 (36.4)		
Positive	768 (48.8)	534 (48.5)	234 (49.6)		
Surgery				0.944	0.624
No	189 (12.0)	133 (12.1)	56 (11.9)		
Other	178 (11.3)	130 (11.8)	48 (10.2)		
Total thyroidectomy	1206 (76.7)	838 (76.1)	368 (78.0)		
Radiation					
No	436 (27.7)	312 (28.3)	124 (26.3)	0.704	0.401
Yes	1137 (72.3)	789 (71.7)	348 (73.7)		
Chemotherapy				0.042	0.837
No	1453 (92.4)	1018 (92.5)	435 (92.2)		
Yes	120 (7.6)	83 (7.5)	37 (7.8)		

RN, positive, regional nodes metastases.

**FIGURE 3:**
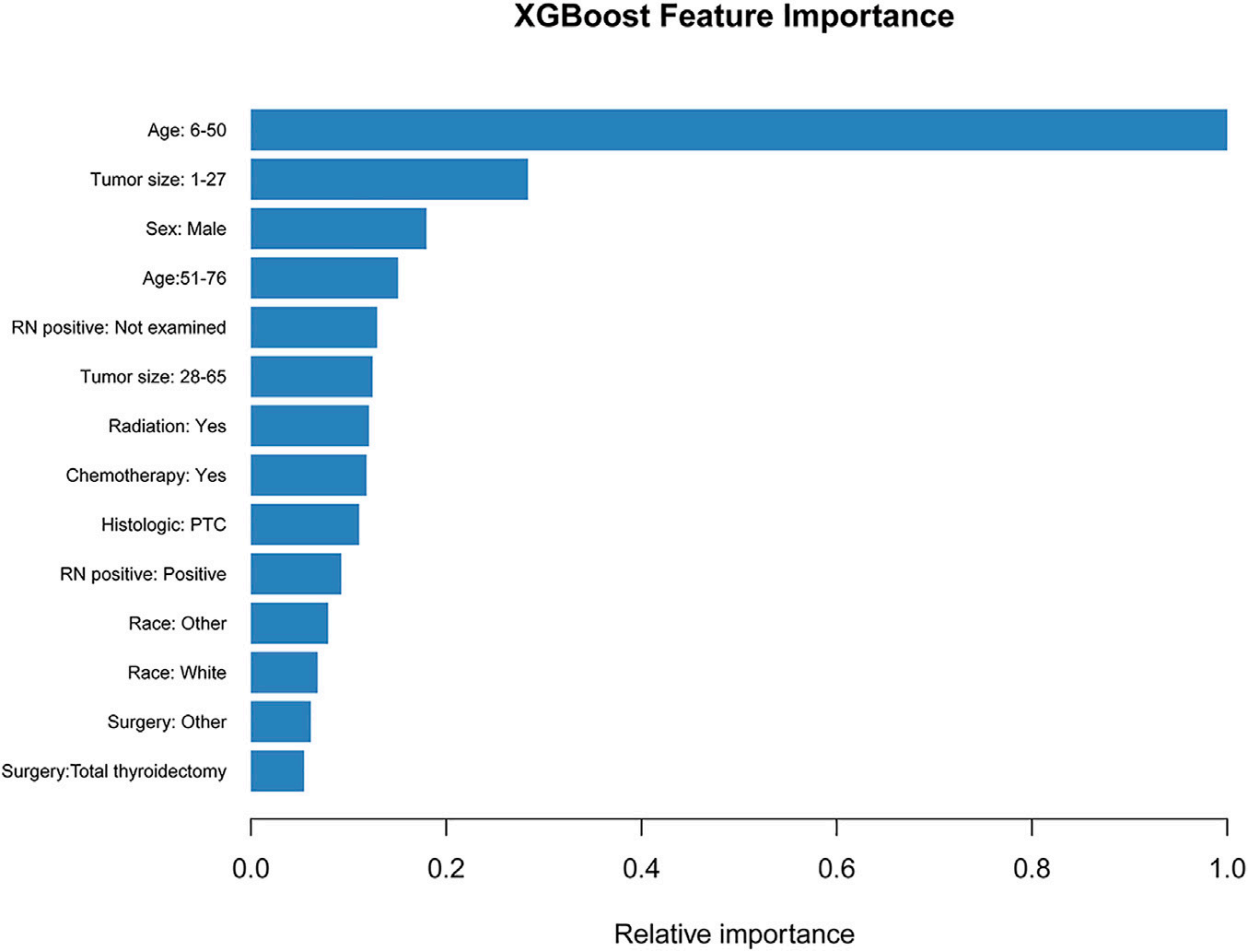
The XGB model was used to calculate the importance of each feature. The bar chart depicts the relative significance of the variables.

Unlike associated studies (Hou et al., 2020; Wei et al., 2021), categorical variables were included in the prediction model as “dummy variables”, age: >76 years, sex: female, race: black, histological type: FTC, tumor size: >65 mm, surgery: no, radiation: no, chemotherapy: no, and RN_positive negative as a control. The XGB algorithm identifies the importance of features based on the magnitude of the gain value obtained for each variable (relative importance scores out of 100), with higher values indicating greater importance to the predicted target. The variables with the highest scores are: age: 6–50 years (39points), tumor size: 1–27 mm (11 points), sex: male (7 points), age: 51–76 years (6 points), RN_positive: not examined (5 points), tumor size: 28–65 mm (5 points), and radiation: yes (5 points) **(** Figure 3 **)**. These variables were included in the LR, SVM, RF models, along with the XGB model for performance testing.

### Model Performance

The optimal model performance parameters were determined after several validations and debugging. To assess the ability of the XGB, SVM, LR, and RF models to identify the OS of patients with 10-years DTCDM, the training and validation sets were plotted, and the AUC was calculated.

**FIGURE 4:**
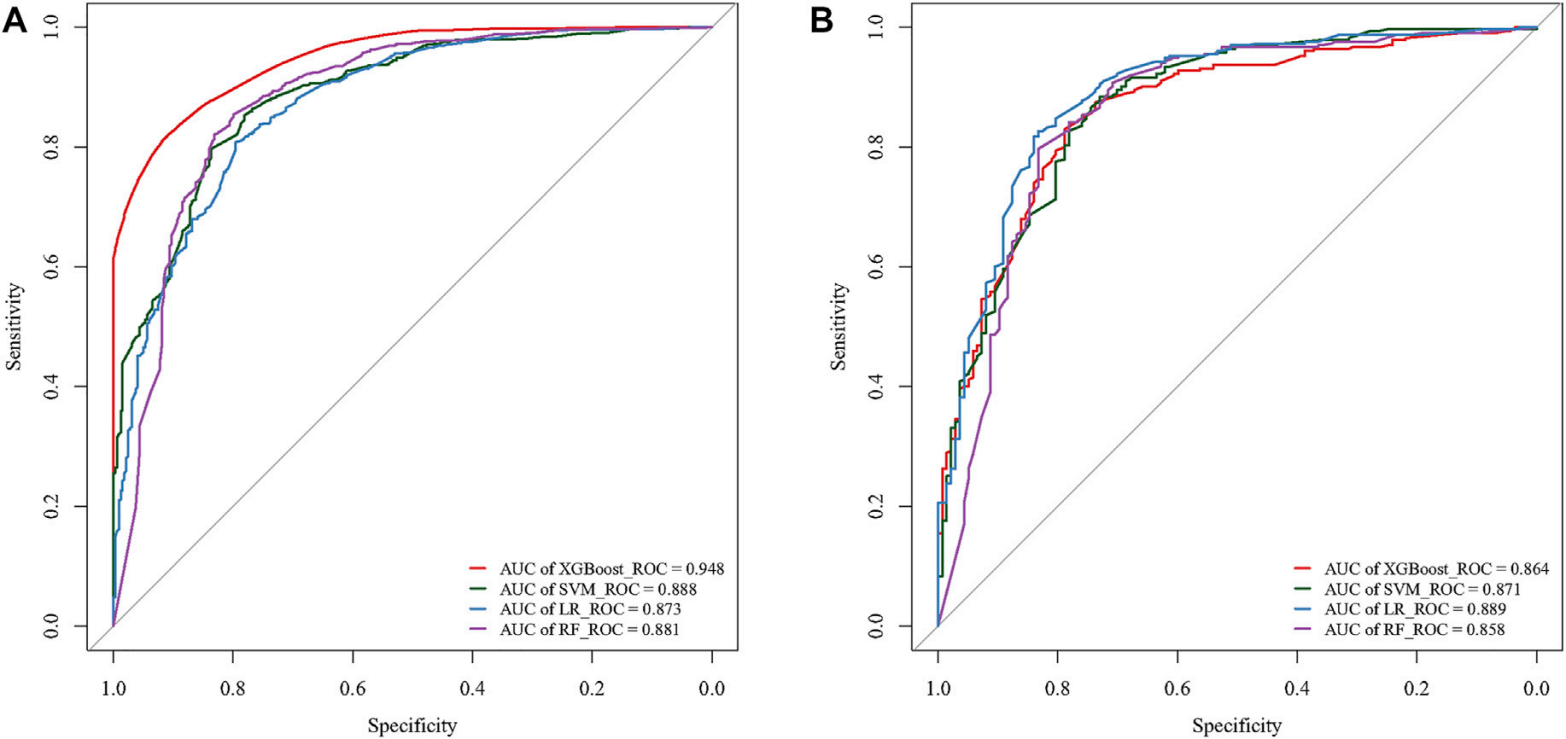
ROC curves of the models for the training **(A)** and test **(B)** cohorts.

**FIGURE 5:**
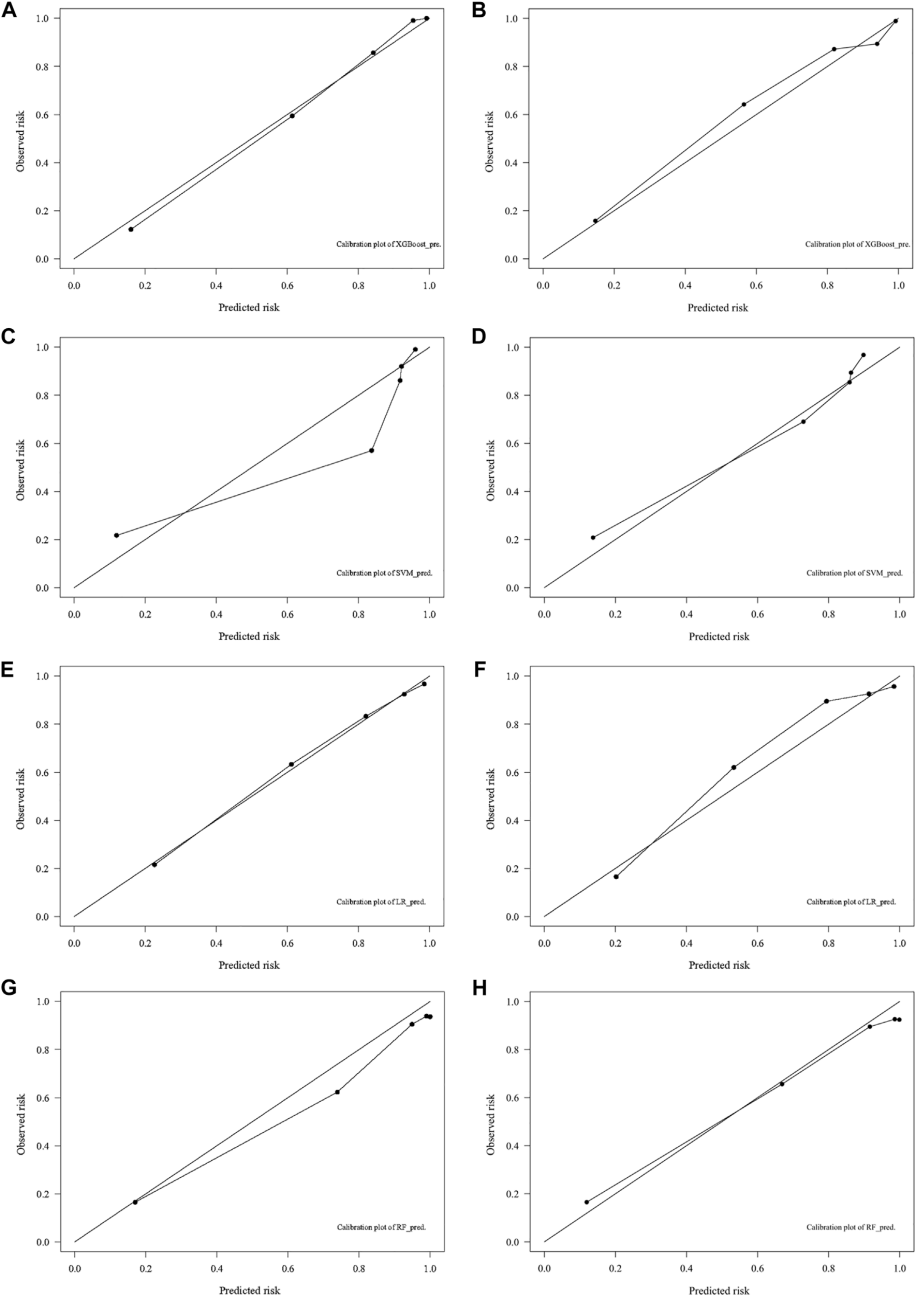
Calibration plots for predicting 10-years DTC development with DM in the training **(A,C,E,G)** and test **(B,D,F,H)** cohorts. DTC: differentiated thyroid cancer, DM: distant metastasis, XGB: XGBoost, SVM: support vector machines, RF: random forest, LR: logistic regression.

**FIGURE 6:**
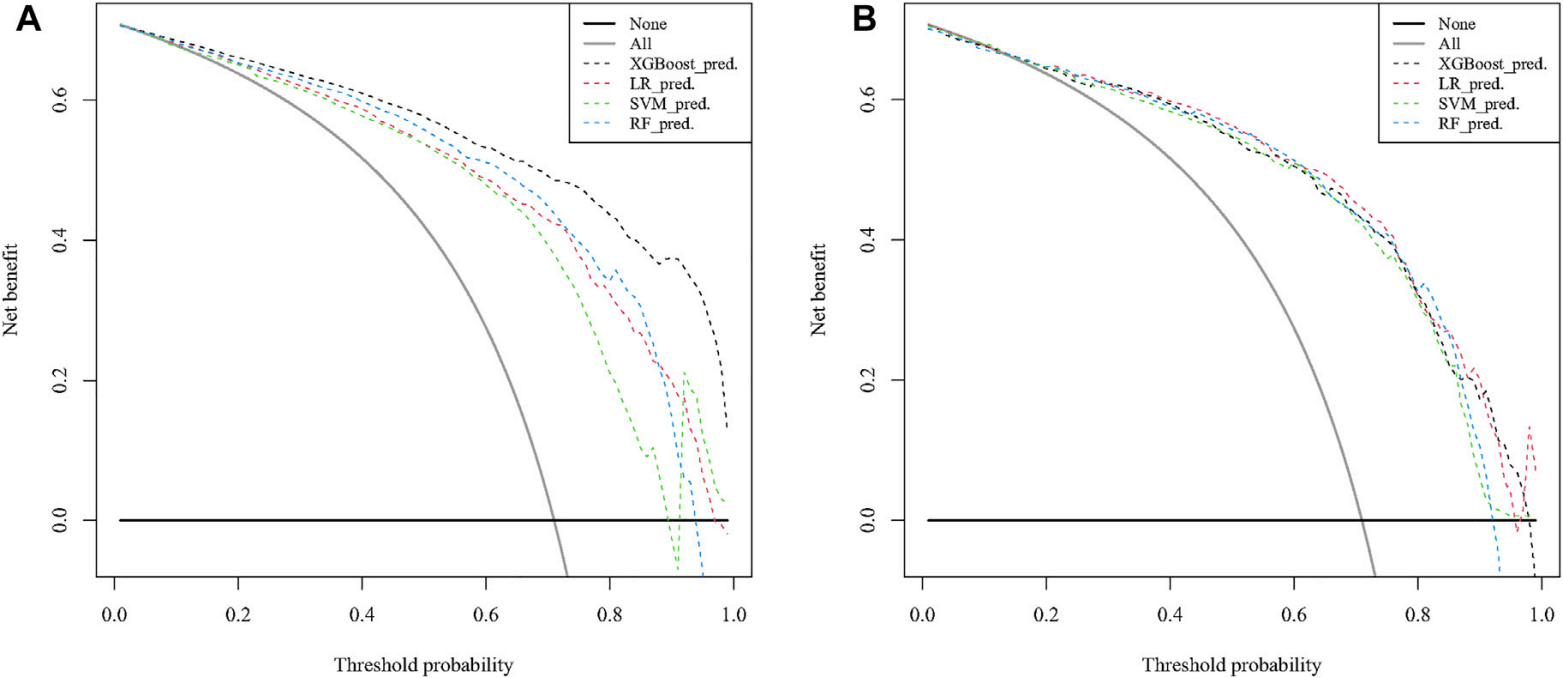
Decision curve analysis to predict 10-years DTC development with DM in the training **(A)** and test **(B)** cohorts. DTC: differentiated thyroid cancer, DM: distant metastasis, XGB: XGBoost, SVM: support vector machines, RF: random forest, LR: logistic regression.

In the training cohort, the AUC of the XGB model was 0.948, higher than the AUC of the other models (SVM AUC, 0.888; LR AUC 0.873, RF AUC,0.881), and The AUC of XGB in the test group was 0.864, slightly lower than the AUCs of LR and SVM (0.889 and 0.871) and higher than the AUC of the RF model (0.858) **(** Figure 4 **).** The calibration plots of the 10-years TCDM OS for the training and test sets showed good linear agreement between predicted and actual observations from the XGB model. The XGB and LR models fit better than the SVM and RF models **(** Figure 5). The DCA curves for the XGB, SVM, RF, and LR models were plotted for the training and test cohorts. The *y*-axis of the decision curve represents the net benefit, the decision analysis metric that determines whether the benefits of a particular clinical decision outweigh the harms. Each point on the *x*-axis represents a threshold probability that distinguishes between patients who die and those who survive. The analysis shows that the XGB, LR, RF, and SVM models all achieve a net clinical benefit **(** Figure 6 **)**.

## Discussion

Survival prediction is very difficult in malignancy but important for treatment planning and patient management (Cheon et al., 2016). Compared to the empirical predictions of clinicians, the XGBoost model provides a more reliable option for the 10-years survival status of patients with distant metastases from thyroid cancer. The current study found that XGB had a good predictive value and could help clinicians to develop a rational individualized treatment and management plan. Although thyroid cancer is a relatively slow-growing cancer, once distant metastasis has occurred, the tumor grows exponentially at the location of metastasis, explaining why patients with DM have a worse prognosis (Rajan et al., 2020). While DTC generally has a favorable prognosis, the clinical course can be poor (See et al., 2017). Assessing the survival of patients with DTCDM is thus of great clinical importance.

This study used survival analysis to screen for factors that may affect the OS of patients with DTCDM. Although, the multiple Cox model we developed showed that race is not a prognostic factor for DTCDM patients. One study showed that race does affect the survival of patients with differentiated thyroid cancer and that treatment options need to be specific to different racial groups (Tang et al., 2018). However, Crepeau et al. (Crepeau et al., 2021) found that thyroid cancer prognosis and recurrence did not differ by race following the same surgical approach, especially when all patients receive the same quality of care. It is likely that differences in the prognosis of TC patients by race are the result of social and economic differences between racial groups.

The XGB algorithm is a new type of AI algorithm that is easy to use, efficient and accurate. This algorithm is becoming increasingly popular in the medical field and is now widely used for disease prediction and early diagnosis. The current study used the prognostic variables obtained from survival analysis to develop a prognostic model for DTCDM OS using the XGB algorithm. The model was validated by combining the clinical and non-clinical variables and was shown to be highly effective. Additional factors that may affect OS and provide more clinical information about DTCDM, were also reported. Traditional ML LR also performed well, possibly because of the study data or because LR performs just as well as ML in clinical prediction models (Christodoulou et al., 2019).

Age was the highest-scoring, and thus most significant, variable in the XGB model, Patients <51 years of age had significantly higher OS than patients >80 years of age. The role for age in predicting TC has been confirmed by other studies, with older patients having a poorer prognosis than younger patients (Sampson et al., 2007; Nixon et al., 2012). Compared with the 8th edition of the TNM staging system with a cut-off value of 55 years, the optimal cut-off values for age in this study were 50 and 77 (Amin et al., 2017). This implies that the cut-off value for age may need to be studied in depth for patients with distant metastatic thyroid cancer. In the current study, there was also a significant score for the 51–79 age group. These findings suggest that treatment should be tailored to patients in different age groups.

The results of one study confirm that patients with PTC with distant metastases have a good prognosis after treatment (Sampson et al., 2007). Another study found that sex is a prognostic factor for DTCDM, likely because estrogen production limits cancer progression (Suteau et al., 2021). The current study found that tumor size was an important factor affecting DTCDM OS, with relatively significant scores for both 1–27 mm and 28–65 mm, a finding supported by Nguyen et al. (Nguyen et al., 2018). Han et al. reported that 15–20 mm tumors do not affect the OS of TCDM patients (Han et al., 2017). Unexamined metastases and those localized in the lymph nodes also scored highly. The number of lymph node metastases correlates strongly with the presence of DM while the risk of DM can be assessed based on the number of lymph nodes (Jeon et al., 2016). These findings may help to resolve controversy over the indication of lateral lymph node dissection (Fan et al., 2018). The regional metastatic status of the lymph nodes should be assessed in all patients with DTCDM.

Radiotherapy had a relatively significant score in the OS prognostic model of patients with DTCDM. Studies indicate that radioactive iodine (RAI) treatment is very effective in patients with small metastases, indicating that early diagnosis improves outcomes (Durante et al., 2006; Sampson et al., 2007). Diagnosis of DM and initiation of RAI therapy before overt metastases appear, especially in children and adolescents for whom selective treatment is more appropriate (Sugino et al., 2020). While RAI treatment is beneficial for TC survival, however, high-quality RAI accumulation may increase the risk of secondary tumor mutations and more aggressive disease, thus negatively impacting patient survival (Su et al., 2015; Pasqual et al., 2022). Nies et al. concluded that repeated RAI treatment is unlikely to benefit TC patients and may do more harm than good over their lifespan (Nies et al., 2021). In summary, studies differ on whether and how to treat DTC patients with RAI (Jeon et al., 2016; Lin et al., 2018).

Total thyroidectomy and other surgical procedures were important in the prognostic model. Sampson et al. concluded that a total thyroidectomy should be performed alongside RAI treatment (Sampson et al., 2007). It is also suggested that, where possible, local curative surgery with RAI and thyroid hormone suppression should be performed in patients with DTCDM (Ito et al., 2010). However, the survival benefit of thyroid cancer surgery may vary depending on the site of metastasis (Besic et al., 2016). The current study found that chemotherapy was a strong predictor of OS in patients with DTCDM and a risk factor for OS in survival analysis. Chemotherapy is often administered to patients with large tumors who are no longer candidates for surgery or show iodine resistance. However, a study indicates that chemotherapy is highly toxic and is associated with a poor response rate (Schmidbauer et al., 2017). The high mortality rate of chemotherapy patients may be due to the relative severity of the disease in this group of patients, in addition to the toxic impact of the treatment. Recent studies have shown that targeted therapies such as tyrosine kinase inhibitors (TKIs) offer high survival rates and that patients may have a better outcome if targeted treatments are combined with chemotherapy (Kraeber-Bodéré et al., 2010; Carling and Udelsman, 2014; Viola et al., 2016; Lorusso et al., 2021).

Although the predictive model used in this study had a good performance, there were some limitations. First, the study relied on regression data and some samples with missing information were removed, which may have biased the model. Second, outcome data for individuals receiving targeted therapies were not included in the sample, which may have made the prediction model less comprehensive. Finally, more work needs to be done to explain the predictive efficacy of ML versus traditional statistical methods.

## Conclusion

This study analyzed the clinical characteristics and prognosis of patients with DTCDM and constructed prognostic models using four machine learning methods. The XGB model was effective at predicting the 10-years OS of patients with DTCDM and may help clinicians to make more accurate and personalized clinical decisions. This is particularly important to improve the long-term prognosis of high-risk patients.

## Data Availability

The datasets presented in this study can be found in online repositories. The names of the repository/repositories and accession number(s) can be found in the article/Supplementary Material.
