# Supplementary figures and images for: A Predictive Model for the 10-year Overall Survival Status of Patients With Distant Metastases From Differentiated Thyroid Cancer Using XGBoost Algorithm-A Population-Based Analysis
Source: Front Genet. 2022 Jul 8;13:896805. doi: 10.3389/fgene.2022.896805 (PMC9305066; doi:10.3389/fgene.2022.896805)

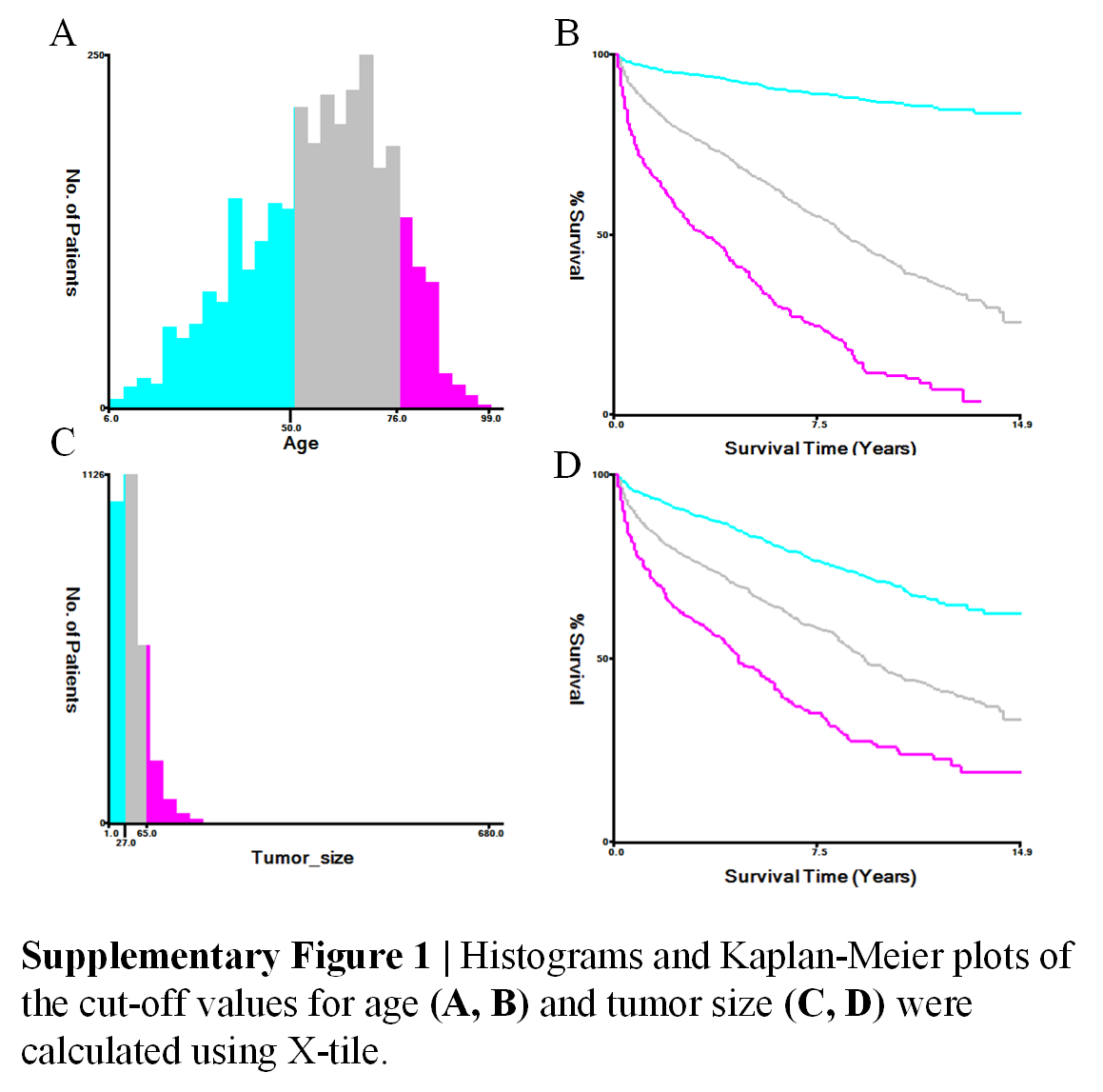

Supplement: Supplementary file 1 [file Image1.TIF]
